# Toward Smart VR Education in Media Production: Integrating AI into Human-Centered and Interactive Learning Systems

**DOI:** 10.3390/biomimetics11010034

**Published:** 2026-01-04

**Authors:** Zhi Su, Tse Guan Tan, Ling Chen, Hang Su, Samer Alfayad

**Affiliations:** 1 Artificial Intelligence Laboratory, School of Design and Art, Changsha University of Science and Technology, Changsha 410114, China; 2 Science, Technology, Engineering, and Mathematics (STEM) Research Group, Faculty of Creative Technology and Heritage, Universiti Malaysia Kelantan, Bachok 16300, Kelantan, Malaysia; 3 College of Engineering and Design, Hunan Normal University, Changsha 410081, China; chenling81@hunnu.edu.cn; 4 The IBISC Laboratory, University of Evry Paris-Saclay, University of Paris-Saclay, 91000 Evry, France

**Keywords:** smart VR education, artificial intelligence, media production training, human-centered design, interactive learning environments

## Abstract

Smart virtual reality (VR) systems are becoming central to media production education, where immersive practice, real-time feedback, and hands-on simulation are essential. This review synthesizes the integration of artificial intelligence (AI) into human-centered, interactive VR learning for television and media production. Searches in Scopus, Web of Science, IEEE Xplore, ACM Digital Library, and SpringerLink (2013–2024) identified 790 records; following PRISMA screening, 94 studies met the inclusion criteria and were synthesized using a systematic scoping review approach. Across this corpus, common AI components include learner modeling, adaptive task sequencing (e.g., RL-based orchestration), affect sensing (vision, speech, and biosignals), multimodal interaction (gesture, gaze, voice, haptics), and growing use of LLM/NLP assistants. Reported benefits span personalized learning trajectories, high-fidelity simulation of studio workflows, and more responsive feedback loops that support creative, technical, and cognitive competencies. Evaluation typically covers usability and presence, workload and affect, collaboration, and scenario-based learning outcomes, leveraging interaction logs, eye tracking, and biofeedback. Persistent challenges include latency and synchronization under multimodal sensing, data governance and privacy for biometric/affective signals, limited transparency/interpretability of AI feedback, and heterogeneous evaluation protocols that impede cross-system comparison. We highlight essential human-centered design principles—teacher-in-the-loop orchestration, timely and explainable feedback, and ethical data governance—and outline a research agenda to support standardized evaluation and scalable adoption of smart VR education in the creative industries.

## 1. Introduction

The convergence of virtual reality (VR) and artificial intelligence (AI) is driving a paradigm shift in the design of educational environments [1,2], particularly in domains that require experiential, creative, and technically intensive learning. Media production, encompassing television program creation, video editing [3,4,5], visual effects, and broadcast direction, represents a field in which practical proficiency, aesthetic sensibility, and procedural expertise are cultivated not only through theoretical instruction but also through active, situated engagement. Traditional media education often depends on access to physical studios, high-end equipment, and instructor-led demonstrations—resources that are logistically constrained, costly to scale, and limited in their capacity to provide real-time, individualized feedback. In contrast, VR technologies have emerged as powerful tools for simulating professional-grade production environments, enabling learners to engage in immersive [6], interactive, and visually rich scenarios for mastering complex tasks such as camera control, lighting composition, chroma keying, and nonlinear editing. These virtual environments expand access to hands-on experience and offer novel modalities for situated cognition [7,8,9], procedural training, and creative exploration.

However, immersion alone does not ensure effective learning. While VR can recreate realistic production environments, it generally lacks the ability to adapt instruction to individual learner needs, interpret behavioral cues, or provide timely pedagogical feedback. These limitations reduce its effectiveness for complex, multistep tasks such as sequencing shots, managing visual continuity, or coordinating multi-role studio workflows.

To address these gaps, recent research increasingly incorporates AI-driven mechanisms that enhance adaptivity, behavioral interpretation, and affect-aware feedback. Human-centered, interactive learning frameworks—often implemented as Human-in-the-Loop (HITL) environments—apply these AI capabilities to create continuous feedback cycles. In such systems, learners influence the system through their actions, and the system, in turn, adapts to their needs. This bidirectional loop enables fine-grained adjustments to task difficulty, learning pace, interaction modality, and feedback timing. These features are particularly valuable in media production, where learners must repeatedly refine aesthetic choices, manage expressive intent, and coordinate complex visual decisions under time pressure.

In parallel, AI advances have introduced capabilities that extend learning systems far beyond static content delivery. Learner modeling techniques can infer cognitive and affective states from multimodal data streams [10,11], including gaze, posture, speech, and interaction patterns. Reinforcement learning and adaptive sequencing can personalize learning trajectories to align with evolving skill levels. Generative AI, encompassing large language models [12,13,14] and diffusion-based visual synthesis, enables the dynamic creation of instructional scenarios, dialogue scripts, and context-aware visual assets tailored to specific learning goals. Emotion recognition and affective computing allow systems to detect states such as frustration, fatigue, or high engagement in real time, enabling timely and personalized interventions. When integrated into VR-based environments, these AI-driven capabilities form the foundation of smart VR education platforms [15,16] that merge immersive realism with adaptive, responsive pedagogy.

**Figure 1:**
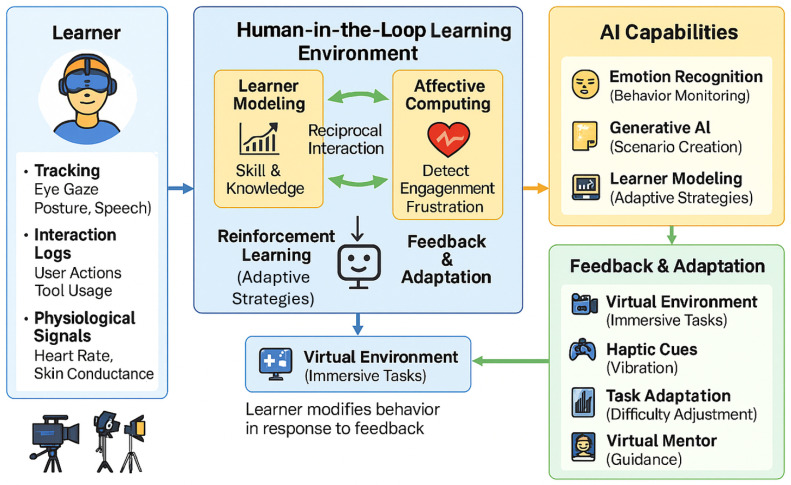
Conceptual framework of Smart VR Education illustrating multimodal sensing, AI-driven learner modeling, and adaptive feedback processes relevant to media production learning.

Despite their promise, the combined application of AI and VR in media production education remains underexplored. While VR has been studied extensively in technical training contexts and AI in intelligent tutoring systems [17], few works systematically address their integration to create adaptive, interactive, and domain-specific learning experiences. The current research landscape is fragmented, often focusing narrowly on visual simulation, adaptive instruction, or emotional engagement without a holistic framework that reflects the complexity of real-world media production workflows. Moreover, existing reviews tend to generalize across diverse disciplines, overlooking the unique pedagogical, cognitive, and affective demands of television and media training [18,19]. To address these gaps, this review synthesizes recent developments at the intersection of VR, AI, and interactive learning, with a particular focus on media production education. It maps key technological trajectories, categorizes system features across adaptation, feedback, and learner engagement, and examines exemplary implementations that demonstrate emerging forms of educational innovation. The aim is to identify both opportunities and challenges in designing Smart VR systems that are immersive, pedagogically intelligent, emotionally responsive, and contextually aligned with creative media practices. Grounded in a human-centered perspective, this work contributes to broader discussions on the future of digital learning environments and provides practical insights for researchers, educators, and developers seeking to transform media education through intelligent immersive technologies. As illustrated in Figure 1, the framework guiding this review integrates multimodal sensing, AI-driven learner modeling, and adaptive feedback processes to support personalized learning in media-production contexts.

This review makes three primary contributions to the field of AI-augmented VR for media production education:

- Structured synthesis: A systematic scoping review of 94 studies (2013–2024) spanning immersive platforms, AI components, and interaction modalities;
- Human-centered perspective: Connecting learner modeling, adaptive feedback loops, and teacher-in-the-loop orchestration to concrete media-production workflows;
- Evaluation toolkit: Identifying common outcome domains and assessment instruments to support comparability across future studies.

## 2. Review Methodology

This study adopts a systematic scoping review approach to identify, evaluate, and synthesize scholarship at the intersection of virtual reality (VR), artificial intelligence (AI), human-centered learning, and media production education. The review is reported in accordance with PRISMA 2020 guidance for transparent reporting of study identification, screening, eligibility, and inclusion [20]. The completed PRISMA checklist is included in the Supplementary Files. Given the substantial heterogeneity in study designs, interventions, and outcome measures, we synthesized the evidence narratively and complemented it with descriptive statistics.

### 2.1. Search Strategy

We searched five major scholarly databases: Scopus, Web of Science, IEEE Xplore, ACM Digital Library, and SpringerLink, for publications from 2013 through 2024 (inclusive). Eligible document types were peer-reviewed journal articles, full-length conference papers, and scholarly book chapters in English.

To capture both technological and pedagogical dimensions, search strings were constructed with Boolean logic and grouped into four thematic clusters:

1. “virtual reality” OR “immersive learning” OR “3D simulation”;
2. “artificial intelligence” OR “adaptive learning” OR “machine learning”;
3. “human-centered” OR “human-in-the-loop” OR “interactive systems”;
4. “media production” OR “television education” OR “video editing training”.

A representative canonical query was:

"virtual reality" AND "artificial intelligence" AND
("human-centered" OR "human-in-the-loop") AND
("media production" OR "television" OR "broadcasting" OR "video editing")

Records were exported from each database and de-duplicated using an automated reference-management workflow, followed by manual verification prior to screening.

### 2.2. Inclusion and Exclusion Criteria

Studies were included if they met all of the following:

1. Described VR-based or AI-enhanced educational systems;
2. Addressed media production or closely related creative-technology domains (e.g., studio operations, directing, cinematography, editing, VFX, audio, broadcasting);
3. Incorporated human-centered and/or interactive design (e.g., learner modeling, multimodal interaction, teacher-in-the-loop orchestration);
4. Appeared in peer-reviewed venues (journals, conferences, or scholarly book chapters);
5. Reported empirical findings, technical prototypes, or conceptual frameworks relevant to intelligent immersive education.

Studies were excluded if they:

1. Applied VR or AI outside education or without an educational intent (e.g., entertainment, clinical therapy);
2. Focused solely on conventional e-learning without immersive/interactive features;
3. Lacked sufficient detail on system design or learner interaction mechanisms;
4. Were not available in full text or were published in languages other than English.

### 2.3. Screening and Selection Process

**Figure 2:**
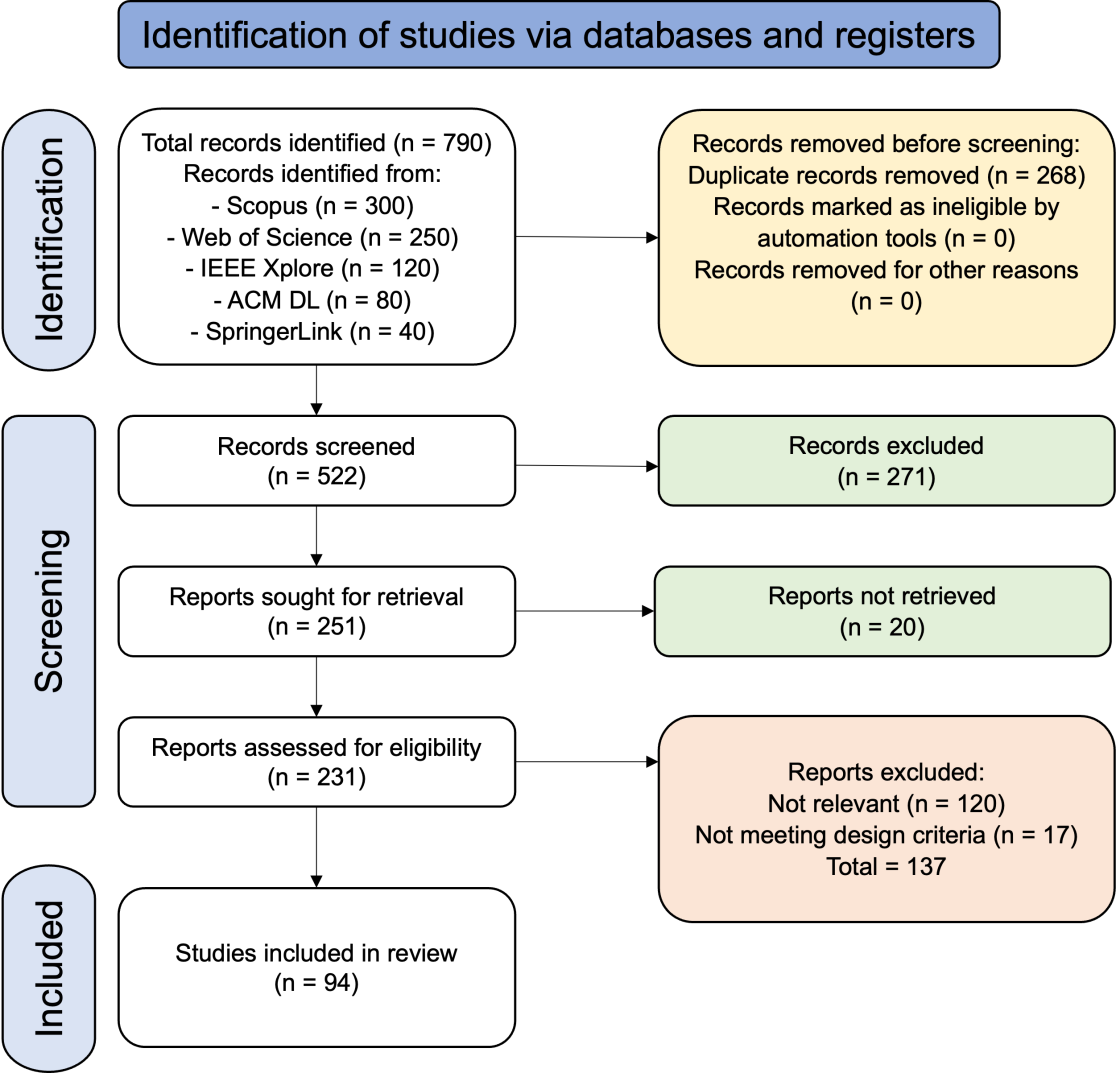
PRISMA 2020 flow of identification, screening, eligibility, and inclusion for English, peer-reviewed records (2013–2024) across Scopus, Web of Science, IEEE Xplore, ACM Digital Library, and SpringerLink. Numbers correspond to Section 2.

The initial search retrieved 790 records: Scopus (n = 300), Web of Science (n = 250), IEEE Xplore (n = 120), ACM Digital Library (n = 80), and SpringerLink (n = 40). After de-duplication, 268 records were removed, leaving 522 unique entries for title/abstract screening. At this stage, 271 records were excluded as out of scope, leaving 251 reports sought for retrieval. We were unable to retrieve 20 full-text reports, resulting in 231 reports assessed for eligibility. In total, 137 reports were excluded at full-text assessment—irrelevant scope (n = 120) and inadequate study design (n = 17)—resulting in 94 studies included in the final synthesis. These figures correspond to the PRISMA flow diagram in Figure 2 and follow PRISMA 2020 reporting [20].

Title/abstract and full-text screening were conducted independently by two reviewers; disagreements were resolved by discussion and, when necessary, adjudication by a third reviewer in line with PRISMA 2020.

### 2.4. Data Extraction and Coding

Data extraction followed a structured codebook. The extracted variables included: bibliographic information; participant or sample characteristics; VR hardware and interaction modalities (e.g., gesture, gaze, voice, haptics); AI components (e.g., learner modeling, adaptive sequencing, affect sensing, NLP/LLM assistants); instructional contexts (e.g., studio workflows, editing, broadcasting); evaluation instruments (e.g., usability, presence, workload, affect, collaboration, scenario-based performance); data sources (interaction logs, eye tracking, biosignals); and key findings.

Two reviewers piloted the codebook on a small subset of studies before applying it to the full dataset. Coding disagreements were discussed and resolved by consensus. In addition to descriptive coding, we conducted a thematic analysis to cluster studies into several recurring categories, including adaptive learning, emotion-aware systems, real-time feedback, creative task simulation, and multimodal interaction.

### 2.5. Quality Appraisal

Methodological quality was assessed using criteria commonly applied in EdTech and HCI research. These criteria covered study design clarity, sample adequacy, measurement validity and reliability, bias and confounding control, analytical transparency, reproducibility, and ecological validity. Each study was evaluated independently by two reviewers, with discrepancies resolved by discussion. Quality judgments informed the interpretive weight given to individual studies but did not affect inclusion.

### 2.6. Data Synthesis and Reporting

Given the heterogeneity of interventions and outcomes, we present a narrative synthesis complemented by descriptive statistics; no meta-analysis was attempted. We report counts and proportions of AI components and interaction modalities, summarize commonly used evaluation instruments, and relate design choices to educational outcomes where comparable. No new human participant data were collected by this review. To balance transparency and readability, study characteristics are summarized using aggregated distributions across media-production subdomains, VR system types, AI components, and evaluation dimensions, rather than listing all 94 studies individually. These aggregated distributions underpin all corpus-level statements reported in the Results.

### 2.7. Core Components of the VR-Based Training Framework

**Figure 3:**
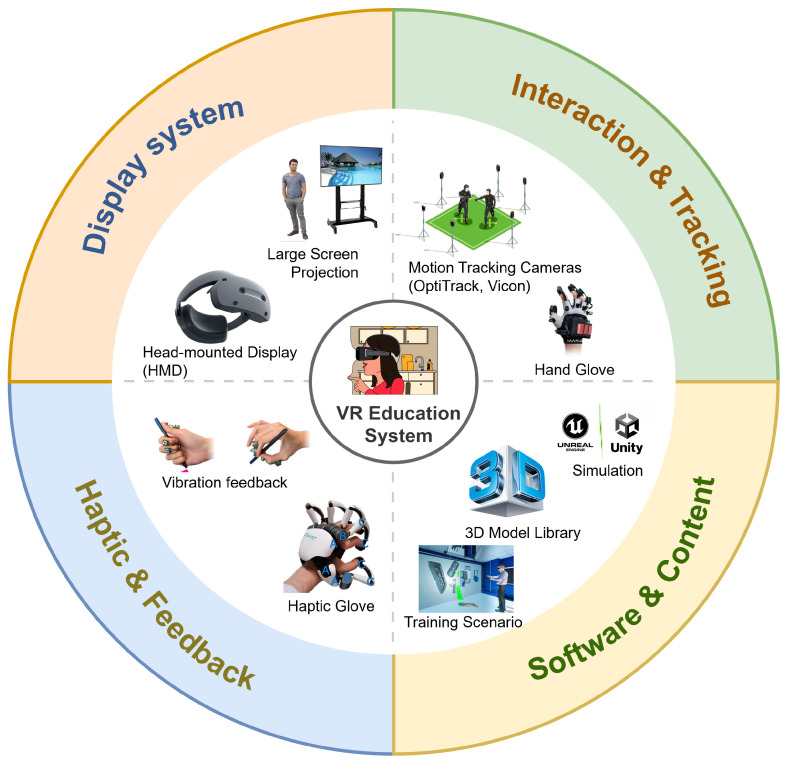
Core components of a VR-based training framework for media production.

To support later analysis, we also mapped the common system architectures described in the included studies. As shown in Figure 3, VR-based training systems in media production typically comprise four functional modules:

- Display system: Head-mounted displays (HMDs), large projection screens, and CAVE-like environments that simulate professional studio settings.
- Interaction and tracking module: Motion capture systems, VR controllers, gesture-recognition gloves, and optical or inside-out tracking for naturalistic interaction with virtual tools.
- Haptic and feedback module: Haptic pens, force-feedback gloves, and vibration actuators that provide tactile cues and enhance task realism.
- Software and content module: 3D modeling tools, game engines (e.g., Unity, Unreal Engine), and scenario-specific training environments.

Together, these modules enable immersive, interactive, and feedback-rich simulations that closely mirror real-world media production workflows.

## 3. Background and Conceptual Foundations

This section provides a foundational understanding of the key domains underlying Smart VR education [21] in media production [22]. We begin by outlining the educational context and specific learning needs of media production disciplines, followed by an exploration of human-centered learning principles in virtual reality environments [23]. Finally, we examine the role of artificial intelligence in enabling adaptive, interactive, and emotionally responsive learning systems [24].

### 3.1. Media Production Education: Needs and Characteristics

Media production education encompasses a diverse set of technical and creative skills, including visual storytelling, camera work, directing, nonlinear editing, motion graphics, and visual effects integration. These competencies require learners not only to master complex software tools and production workflows but also to develop an artistic sensibility and narrative fluency. In contrast to purely theoretical instruction, effective training in this domain often involves iterative experimentation [25], real-time decision-making, and feedback-driven refinement, mirroring the high-stakes, collaborative nature of real-world media environments.

Pedagogically, this domain presents unique challenges. It requires a delicate balance between fostering individual creativity and imparting procedural knowledge. Learners must understand both the aesthetic principles of composition, color, and rhythm, and the technical constraints of production pipelines, rendering engines, and editing interfaces [26]. Furthermore, the collaborative aspects of media production—such as directing teams, managing timelines, and coordinating across roles—introduce additional layers of complexity. Traditional classrooms and computer labs are often ill-suited to support these multifaceted demands due to limitations in resources, scalability, and real-time experiential feedback. These conditions underscore the need for immersive, interactive platforms that allow learners to practice media production tasks in realistic yet flexible environments.

### 3.2. Human-Centered Learning in Virtual Reality

Human-centered learning in virtual reality [27] emphasizes the active role of learners in shaping their own educational experience through real-time interaction, control, and contextual relevance. Unlike content-driven instructional models, human-centered approaches prioritize learner engagement, autonomy, and adaptability, recognizing that effective learning emerges from meaningful interaction with the environment and with learning tasks [28]. In the context of VR, this translates into systems that allow users to navigate spatial environments, manipulate objects, receive real-time feedback, and engage with learning materials at their own pace and sequence.

**Figure 4:**
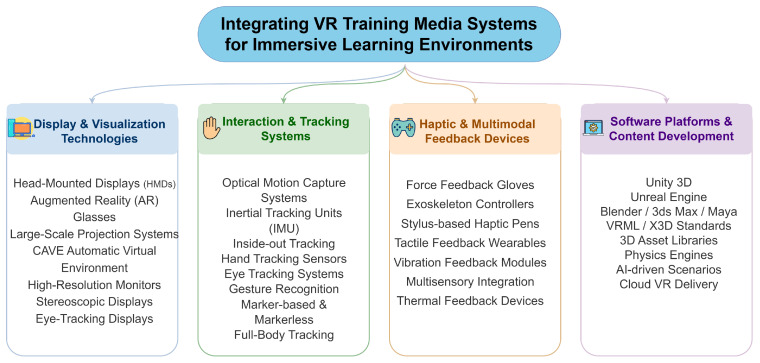
Integration framework for VR training media systems. Four layers—display & visualization, interaction & tracking, haptic/multimodal feedback, and software/content—provide the technological substrate for immersive training tasks in studio workflows.

An integrated framework for VR-based training media systems is shown in Figure 4. The framework consists of four core components: display and visualization technologies; interaction and tracking systems; haptic [29,30] and multimodal feedback devices [31]; and software platforms and content-development tools.

Display and visualization technologies. This category includes the hardware that delivers immersive visual experiences. Common solutions include head-mounted displays (HMDs), augmented reality (AR) glasses, large-scale projection systems, CAVE systems (Cave Automatic Virtual Environment), high-resolution monitors, stereoscopic displays, and eye-tracking-enabled displays for adaptive rendering [32].

Interaction and tracking systems. These systems support accurate user input and spatial awareness in VR. They encompass optical motion-capture systems, inertial tracking units (IMUs), inside-out tracking, hand-tracking sensors, eye-tracking systems [33,34], gesture-recognition methods, marker-based and markerless tracking, and full-body tracking solutions.

Haptic and multimodal feedback devices. This category focuses on providing tactile and force feedback. Representative technologies include force-feedback gloves, exoskeleton-based controllers, stylus-based haptic pens, wearable tactile devices, vibration modules, multisensory feedback integration, and thermal feedback devices. Such mechanisms reinforce equipment-handling skills and enhance task realism.

Software platforms and content development. This component covers the toolchains used to create, manage, and deliver VR training content. Key technologies include game engines such as Unity and Unreal Engine; 3D modeling tools like Blender, 3ds Max, and Maya; standardized content formats such as VRML/X3D; 3D asset libraries and physics engines; and emerging tools for AI-driven scenario generation and cloud-based VR content delivery.

Together, these four components form a comprehensive technological ecosystem that supports the design, development, and deployment of VR-based training media systems.

**Figure 5:**
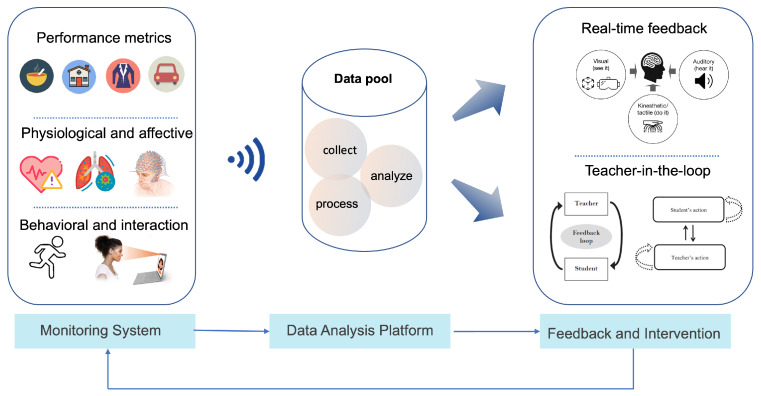
Monitoring & feedback architecture: performance, physiological/affective, and behavioral signals are collected and analyzed in a data pool to drive real-time feedback. A teacher-in-the-loop channel enables timely orchestration and task adaptation within the same pipeline.

Figure 5 presents the architecture of the VR training monitoring and feedback system. The monitoring module collects data on three primary dimensions: performance state (e.g., task completion and efficiency), physiological state (e.g., heart rate, respiratory patterns, neural activity), and behavioral state (e.g., movement patterns and gaze behavior). All data are transmitted to a centralized data pool for collection, processing, and analysis. The analyzed results feed into the feedback and intervention module, which provides real-time feedback through visual, auditory, and kinesthetic/tactile channels, enhancing user engagement and learning efficiency. Additionally, a teacher-in-the-loop mechanism enables adaptive guidance, where instructors dynamically adjust tasks and provide targeted interventions based on the learner’s actions and progress, thus creating a closed-loop system for optimized immersive training [35,36,37].

Theoretical foundations for human-centered VR learning, as shown in Table 1, draw on multiple disciplines. Embodied cognition theory posits that cognitive processes are deeply influenced by the body’s interactions with the environment, suggesting that spatial movement, gesture, and physical positioning can enhance conceptual understanding. Flow theory [38] describes the optimal state of engagement where challenge and skill are balanced, motivating learners to persist and improve. The concept of presence refers to the subjective sense of “being there” in a virtual space, which is essential for immersion and engagement [39]. Learner control theories emphasize the importance of giving learners the ability to make meaningful choices that affect their experience, thereby increasing motivation and agency. Affective engagement models highlight how emotional responses—such as curiosity, frustration, or excitement—mediate attention and learning outcomes.

In practical terms, these principles manifest in VR systems through features such as customizable learning paths, multimodal interfaces (e.g., gesture, voice, gaze), task-based scaffolding, and adaptive feedback mechanisms. Human-centered design ensures that such systems are not only technologically sophisticated but also pedagogically effective, providing intuitive and emotionally resonant learning experiences. In media production education, where timing, aesthetics, and procedural fluency are tightly interwoven, these human-centered affordances are especially critical.

### 3.3. Artificial Intelligence in Learning Environments

Artificial intelligence plays an increasingly central role in the evolution of digital learning environments, offering a wide array of tools to enhance personalization, adaptability, and efficiency. In the context of VR-based learning, AI functions as the cognitive engine that allows systems to sense, interpret, and respond to learner behaviors in real time. Key AI technologies relevant to education include learner modeling, reinforcement learning, adaptive content sequencing, and emotion recognition.

Learner modeling involves building dynamic representations of a learner’s knowledge state, preferences, emotional disposition, and performance over time. These models can be derived from multimodal input such as task completion data, interaction logs, physiological signals, and behavioral cues. Reinforcement learning enables systems to optimize instructional strategies by continuously updating policies based on feedback signals and observed performance [47,48]. Adaptive sequencing techniques determine the optimal order and complexity of learning tasks to match individual learners’ progression and needs. Emotion recognition systems leverage computer vision, speech analysis, or biometric sensors to infer affective states such as confusion, boredom, or engagement, enabling timely pedagogical interventions.

Together, these AI components support dynamic personalization, as shown in Table 2, allowing systems to deliver content that is responsive to the learner’s current state and trajectory. They also enable sophisticated learning analytics, offering insights into patterns of engagement, misconception trends, and long-term progress. Importantly, AI can help reduce cognitive overload by modulating task complexity, pacing, and feedback according to individual learner capacity. This not only enhances the efficiency of learning but also supports greater motivation and resilience by aligning the learning experience with the learner’s cognitive and emotional readiness.

In the context of media production education, AI-driven systems can assist in a range of tasks—from recommending relevant editing tools based on learner behavior to generating simulated production scenarios tailored to specific learning goals. By combining intelligent decision-making with immersive interaction, AI-powered VR systems open new frontiers in training creative professionals, supporting both technical fluency and artistic expression in a personalized and scalable manner.

## 4. Research Landscape: Smart VR Education Systems

**Figure 6:**
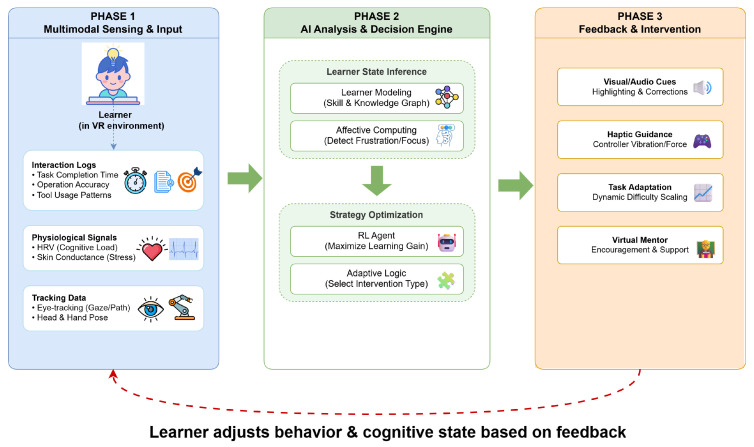
Multimodal learner modeling and adaptive feedback loop in smart VR media production education systems. The system aggregates multimodal data (Phase 1), processes it through an AI engine for state inference and strategy optimization (Phase 2), and delivers real-time interventions (Phase 3), forming a continuous Human-in-the-Loop cycle.

This section explores the current landscape of smart VR-based learning systems designed for media production education. It examines how immersive technologies and artificial intelligence are being leveraged to create adaptive, interactive, and domain-specific learning experiences. The discussion is organized into three thematic subsections: immersive VR platforms, AI-augmented interaction and adaptation, and learner modeling and feedback mechanisms, as shown in Figure 6.

### 4.1. Immersive VR Platforms in Media Production Training

The deployment of immersive virtual reality platforms in media production education represents a major evolution in the pedagogical tools available for training creative professionals. These platforms recreate the spatial, procedural, and technical conditions of real-world media environments, allowing learners to perform practical tasks such as studio directing, camera operation, lighting setup, video editing [49,50,51], and live broadcasting within simulated yet high-fidelity virtual spaces. Unlike traditional instructional methods that rely heavily on observation or passive demonstrations, immersive VR enables direct engagement with production tools and workflows in a hands-on, embodied manner. This form of interaction is particularly advantageous in media education, where spatial awareness, real-time coordination, and procedural execution are fundamental to skill acquisition.

Virtual studio platforms typically model complete production environments, including control rooms, chroma key stages, lighting rigs, and multi-camera setups. Learners are placed in these synthetic spaces and interact with equipment analogs such as virtual switchers, camera tracks, boom mics, and teleprompters. These systems offer high degrees of visual and functional realism, often incorporating advanced physics engines to simulate real-time lighting changes, shadow diffusion, motion blur, and environmental audio effects [52]. Editing simulators expand the affordances of traditional non-linear editing software by allowing learners to navigate timelines, apply transitions, and synchronize audiovisual layers using gestures, eye gaze, or speech input. Such immersive timelines are designed to teach both tool proficiency and aesthetic decision-making by providing real-time feedback on rhythm [53,54], continuity, and pacing. In more advanced applications, VR environments are used for broadcast rehearsal, where students simulate live shows by switching between camera feeds, adjusting audio levels, coordinating cues, and responding to unexpected disturbances, all within time-sensitive scenarios that mirror the pressure of real-world production.

A defining characteristic of these immersive platforms is task fidelity—the extent to which the tasks and workflows presented in the virtual environment mirror those encountered in actual professional settings. This includes not only the look and feel of the tools, but also the constraints learners must navigate, such as limited render times, bandwidth ceilings for live streaming, or the coordination of multiple assets under tight production timelines. Many platforms emulate industry-standard software environments and export real-format deliverables, thus ensuring the transferability of skills acquired in simulation to real practice. Spatial interaction is another cornerstone of these systems. Learners physically move within virtual sets, adjust camera perspectives, manipulate lighting angles, and explore different blocking strategies. Such embodied learning experiences support the development of muscle memory, spatial reasoning, and sensory feedback integration, which are essential for mastering complex production setups.

These platforms are not limited to individual training; several systems support collaborative VR sessions [46] where learners co-inhabit shared environments, assuming different production roles and coordinating as a team. This feature addresses the inherently social and interdependent nature of media work. Moreover, the flexibility of VR allows for repeated rehearsal, rapid scenario changes, and simulated production crises that challenge learners to make decisions under pressure. The richness and adaptability of these immersive environments have already begun to reshape the pedagogical landscape of media production education, yet their full potential is only realized when combined with artificial intelligence systems that enable real-time adaptation, feedback, and personalization—topics explored in the next section [55].

### 4.2. AI-Augmented Interaction and Adaptation

Artificial intelligence adds a new layer of intelligence and adaptability to VR-based learning environments by allowing systems to observe, interpret, and respond to learner behavior in real time. In media production education, where learners face a combination of cognitive, technical, and aesthetic challenges, AI plays a pivotal role in adjusting instructional strategies, scaffolding complex tasks, and maintaining engagement through emotionally responsive feedback. By integrating AI into VR platforms, educators can transform passive simulations into intelligent learning ecosystems capable of tailoring experiences to individual learners’ trajectories and emotional states.

One of the primary functions of AI in this context is the dynamic adjustment of task difficulty and sequencing. Rather than following a fixed curriculum, intelligent systems analyze interaction data such as task accuracy, completion time, attention shifts, and interface usage patterns to infer learner proficiency. Based on this data, the system may choose to modify the complexity of the task, offer additional hints, reduce the number of available tools, or reorder instructional modules to better align with the learner’s current cognitive state. These adaptations can be implemented using various algorithmic approaches. Rule-based systems associate predefined learner behaviors with specific instructional responses, while probabilistic models estimate the likelihood of misunderstanding or disengagement. More sophisticated approaches involve reinforcement learning, where instructional agents learn to select optimal teaching strategies by maximizing long-term learning gains through repeated interaction and feedback. These agents can evolve personalized learning paths that account for non-linear skill development, common in creative domains like video editing or scene direction.

AI also enables the integration of emotion-aware interaction mechanisms. Media production tasks are often emotionally charged, involving subjective judgments, public critique, and time-bound deliverables. Learners may experience stress, frustration, or demotivation, especially when facing unfamiliar software, aesthetic uncertainty, or coordination failures in team-based simulations. Emotion-sensitive AI systems leverage multimodal inputs such as facial expressions, gaze tracking, voice inflection, and physiological data to infer affective states. Once an emotional shift is detected—whether it be fatigue, confusion, or heightened engagement—the system can adjust its behavior accordingly. For instance, signs of cognitive overload may prompt the system to pause task progression, suggest a reflective interlude, or present visual summaries of progress to restore orientation and motivation. Alternatively, periods of high engagement can be leveraged to introduce more complex challenges or open-ended creative tasks.

The integration of natural language processing further expands the adaptability of these systems by enabling conversational interfaces within the VR environment. Learners can query the system for clarification, request feedback on their creative decisions, or engage in reflection dialogues about their performance. For example, a student might ask why a lighting setup appears flat or why a transition feels abrupt, and the system could generate context-specific suggestions based on aesthetic principles or technical parameters. This dialogic interaction not only supports knowledge construction but also simulates the presence of a mentor, which is particularly valuable in domains where expert critique shapes learning.

By embedding AI-driven adaptability and emotional sensitivity into VR environments, educational systems become more than passive representations of professional settings. They evolve into interactive, supportive, and context-aware spaces that mirror the complexity of human learning. For media production education, where the interplay of cognition, emotion, and creativity is fundamental, such intelligent systems offer transformative potential in developing learners’ confidence, resilience, and creative autonomy.

### 4.3. Learner Modeling and Feedback Loops

At the heart of adaptive virtual reality (VR) learning systems lies the capability to continuously observe, interpret, and respond to learner behavior. Learner modeling refers to the construction of computational representations that capture a learner’s current knowledge, cognitive state, affective condition, behavioral tendencies, and learning trajectory. These models form the foundation upon which AI-driven personalization and feedback strategies are built. In the context of media production education, where tasks are complex, iterative, and frequently subjective, accurate and dynamic learner modeling is essential for delivering timely, relevant, and meaningful instructional interventions.

Modern learner modeling techniques draw upon multimodal data streams generated through user interaction within immersive VR environments. These include eye-tracking [32,56,57] to assess attention and visual scanning patterns, gesture tracking to evaluate spatial awareness and motor control, posture and head orientation data to detect engagement and situational awareness, and biometric signals such as heart rate variability [58], skin conductance, or EEG for inferring emotional and cognitive load [45]. By integrating these diverse signals, the system can form a composite understanding of the learner’s performance state. For example, frequent gaze shifts combined with prolonged inactivity and elevated heart rate might indicate frustration or confusion during a complex scene editing task. Conversely, smooth and directed gestures with low physiological stress indicators may signify task fluency and cognitive flow.

Once constructed, these models are used to drive feedback loops, wherein system outputs are dynamically adjusted based on interpreted learner states. Feedback can take various forms—visual overlays that guide corrective action, contextual audio prompts, haptic cues through motion controllers [59,60,61], or reflective summaries presented at task completion. Crucially, these feedback mechanisms are not one-way; they are part of a continuous cycle in which the system monitors the learner’s response to the feedback and updates the learner model accordingly. This closed-loop architecture supports real-time adaptation, long-term personalization, and longitudinal tracking of progress across multiple learning sessions. While the feedback loop described above enables real-time adaptation, its learning mechanism can be further specified through the paradigm of Learning with Human Feedback (RLHF). In this formulation, teacher interventions—such as overriding AI-suggested task sequences, adjusting difficulty levels, or providing qualitative assessments of a learner’s VR performance—are treated as structured feedback signals. These signals can be operationalized as reward functions, preference rankings, or policy constraints that guide the continuous update of adaptive components, particularly reinforcement learning–based orchestration modules [62].

Framing teacher input in this way transforms the teacher’s role from a passive supervisor into an active trainer of the AI system, creating a virtuous cycle in which expert pedagogical judgment incrementally aligns system behavior with instructional goals. This perspective mirrors recent RLHF approaches successfully applied to large language models, where human feedback is used to iteratively refine model outputs and decision policies. Importantly, such a design preserves human authority and interpretability while enabling scalable, data-efficient adaptation in complex educational environments [63].

In media production training, such feedback loops are particularly valuable for guiding complex decision-making processes that blend technical precision with creative intent. For instance, when a learner is composing a multicamera sequence, the system might highlight suboptimal angle selection or pacing inconsistencies and provide visual cues or comparative references to professional editing practices. Over time, the system can identify recurring mistakes or stylistic tendencies and adjust its feedback strategy—offering more granular assistance for novices or higher-order critique for advanced users. In directing simulations, feedback might include trajectory corrections for virtual actors, timing alignment for scene transitions, or camera movement smoothness analysis.

Furthermore, these models can inform broader instructional design decisions. Instructors or system designers may use aggregated learner model data to identify common learning bottlenecks, visualize engagement patterns across cohorts, and iteratively refine VR modules. By making learner modeling and feedback both granular and scalable, smart VR systems support not only individualized learning but also evidence-based curriculum development.

## 5. Benefits and Educational Impacts

The integration of artificial intelligence into immersive virtual reality environments for media production education [44] yields a range of pedagogical benefits that extend beyond those offered by either technology in isolation. These intelligent and interactive systems reshape how learners acquire, apply, and reflect upon complex skills, while also introducing structural innovations that make media education more scalable, inclusive, and sustainable.

One of the most significant educational benefits of smart VR systems lies in their capacity to accelerate skill acquisition and enhance procedural fluency. Media production tasks—such as multicamera direction, chroma key compositing, video editing, and real-time effects control—are inherently complex, requiring the coordination of technical precision, aesthetic judgment, and temporal decision-making. Through immersive practice environments that replicate the spatial and procedural conditions of professional studios, learners are able to repeatedly engage with these workflows in a risk-free and self-paced manner. The embodiment of action within VR supports motor memory formation and situational awareness, while intelligent feedback mechanisms ensure that learners not only perform tasks but also understand the rationale behind their choices. As a result, learners develop deeper conceptual models of production processes and gain confidence in executing them under pressure.

Beyond skill proficiency, these systems support the cultivation of personalized learning trajectories—an essential feature in creative domains where learners’ interests, prior experiences, and aesthetic preferences vary widely. AI-driven learner modeling enables systems to adapt content, pace, and instructional strategies based on each user’s evolving profile. A novice might receive step-by-step scaffolded guidance with visual overlays and examples, while a more advanced learner may be offered open-ended challenges and opportunities for experimentation. This level of personalization fosters agency and self-directed learning, aligning instructional pathways with individual goals and learning styles. In contrast to standardized curricula that often constrain creativity, adaptive VR environments encourage exploration and iterative refinement—critical aspects of media practice.

Another important impact of these systems is their ability to sustain motivation and emotional engagement through affect-sensitive feedback. Creative work is emotionally intensive and often involves cycles of doubt, inspiration, frustration, and satisfaction. Traditional instructional systems rarely account for these affective dynamics, leaving learners unsupported during emotional downturns. In contrast, smart VR platforms equipped with emotion recognition capabilities can detect signs of disengagement, confusion, or fatigue and respond with timely, empathetic interventions. These may include motivational messages, reduction of task complexity, or simply suggesting a pause for reflection. Conversely, when high engagement or flow states are detected, the system may escalate task complexity or introduce narrative-based challenges to deepen involvement. By aligning instructional behavior with learners’ emotional rhythms, these systems cultivate a more supportive and responsive educational experience, which has been shown to positively influence persistence and long-term skill development.

In terms of access and scalability, intelligent VR systems offer new possibilities for expanding media production education beyond traditional institutional boundaries. Conventional training in this field often requires physical access to expensive studios, high-end equipment, and expert instructors—resources that are limited and unevenly distributed. VR systems can replicate much of this infrastructure digitally, providing learners with on-demand access to realistic practice environments from remote locations. When coupled with AI-driven personalization and automated feedback, these platforms reduce the dependence on constant instructor supervision, enabling more learners to benefit from high-quality, individualized instruction at lower cost. This democratization of access has particular implications for under-resourced institutions, freelance learners, and geographically dispersed student populations seeking media training without relocating or incurring prohibitive expenses.

Finally, the emergence of smart VR learning environments invites a redefinition of the teacher’s role. Rather than acting solely as content deliverers or technical demonstrators, educators increasingly serve as facilitators, mentors, and co-creators within the learning process. Intelligent systems take on the tasks of monitoring learner behavior, delivering just-in-time feedback, and managing adaptive sequencing, thereby freeing instructors to focus on higher-order pedagogical functions. These may include curating creative challenges, providing interpretive critique, fostering collaborative learning, and guiding students in reflective practice. Instructors also gain access to learner analytics and behavioral insights generated by the system, which can inform assessment, curricular decisions, and targeted interventions. This reconfiguration of instructional roles fosters more meaningful educator–learner interactions and supports a more sustainable model of education that combines automation with human guidance [64].

In sum, the convergence of VR and AI in media production education delivers tangible improvements in skill development, motivation, and personalization, while also enabling more inclusive and scalable training models. By supporting both the cognitive and affective dimensions of learning and redefining the educator’s role [42], these systems contribute to a more flexible, responsive, and human-centered vision of future media education.

## 6. Challenges and Limitations

Despite the promising capabilities of AI-augmented virtual reality systems in transforming media production education, their implementation and widespread adoption face a range of technical, pedagogical, and ethical challenges. These limitations not only constrain current system performance but also shape the research agenda for the next generation of intelligent, immersive educational environments. Addressing these issues is critical for realizing the full potential of human-centered smart VR learning systems.

From a technical perspective, one of the most immediate concerns lies in system latency and rendering performance. Immersive VR environments, particularly those simulating real-time media production tasks, demand high frame rates, low input-to-output delay, and rapid scene updating to maintain perceptual coherence and prevent motion sickness. When such systems are augmented with AI components that process multimodal learner data—including eye tracking [65,66,67], gesture recognition [68,69], physiological signals [70], and voice input—the computational load increases substantially. The fusion of these data streams in real time requires robust synchronization and efficient data pipelines. Delays in processing can disrupt the fluidity of user interaction and diminish the sense of presence, which is essential for effective learning. Furthermore, rendering high-fidelity environments that simulate professional-grade lighting, spatial acoustics, and complex asset animations remains resource-intensive and may not be accessible on standard hardware platforms, particularly for institutions with limited technological infrastructure.

Beyond these performance issues, the integration of artificial intelligence introduces a set of deeper concerns related to transparency, bias, and interpretability. Many adaptive learning systems rely on black-box models—such as deep neural networks or reinforcement learning agents—whose internal logic is difficult to trace or explain. In creative education domains like media production, where learners often wish to understand not only what went wrong but why, the inability of AI systems to provide clear rationales for feedback undermines trust and pedagogical value [71,72]. Biases in training data can also skew system behavior, privileging certain styles, behaviors, or user demographics over others. This is particularly problematic in media education, where diversity of expression and cultural specificity are central to the creative process. Moreover, the collection and storage of granular learner data—especially affective and biometric information—raise concerns about data privacy and consent [73,74]. Without strong governance mechanisms, these systems risk violating learners’ informational autonomy and exposing sensitive data to misuse [75].

In the educational domain, significant challenges remain in aligning intelligent VR systems with sound instructional design [76]. Many prototypes emphasize technical sophistication but lack grounding in established pedagogical theories or curricular frameworks. The design of adaptive pathways often assumes linear learning progressions, which may not align with the iterative and exploratory nature of creative skill development. Moreover, standardized evaluation frameworks for assessing learning outcomes in VR-based environments, as shown in Table 3, are still underdeveloped. Traditional metrics—such as quiz scores [77] or task completion time—fail to capture the richness of affective engagement, collaborative dynamics, or creative quality that characterize media production learning. This gap complicates efforts to validate system effectiveness, compare alternative designs, or justify institutional adoption.

The role of instructors also presents a challenge. While intelligent systems can automate certain aspects of feedback and monitoring, effective integration into real classrooms or studios requires that educators be trained not only in using the technology but also in interpreting system outputs and adapting their teaching strategies accordingly [79]. Many instructors may lack the technical fluency or pedagogical models needed to fully exploit the capabilities of AI-augmented VR platforms. Without sufficient professional development and institutional support, there is a risk that these systems will remain underutilized or be met with resistance.

Ethical and psychological concerns [80] add another layer of complexity. As AI-enhanced VR systems become more sophisticated in interpreting learner behavior and emotion, the line between supportive guidance and manipulation becomes blurred. Over-surveillance—where every movement, decision, and emotional shift is monitored—can create a sense of intrusion, reducing learner autonomy and self-efficacy. Adaptive systems that seek to optimize performance by regulating emotional states may inadvertently shape user behavior in ways that undermine intrinsic motivation or creative risk-taking. There is also the danger of reinforcing normative standards for creativity, potentially discouraging learners who deviate from dominant stylistic conventions. These risks call for careful ethical design principles that prioritize user agency, informed consent, and emotional safety within immersive learning experiences.

In summary, the development and deployment of smart VR education systems are constrained by a constellation of technical bottlenecks, algorithmic uncertainties, pedagogical misalignments, and ethical dilemmas. While these challenges do not negate the transformative potential of such systems, they underscore the importance of interdisciplinary collaboration and critical reflection in their design and implementation. Future research must not only advance the capabilities of VR and AI technologies but also ensure that these tools serve diverse learners equitably, transparently, and responsibly.


Design Principles for Smart VR in Media Production. Teacher-in-the-loop orchestration with overrideable AI actions;Learner modeling to pace task difficulty and reduce overload;Timely, explainable feedback tied to concrete studio workflows;Emotion-aware nudges that respect autonomy and privacy;High-fidelity yet low-latency interaction (low perceived input-to-photon latency, minimizing motion-to-photon delay where feasible);Role-based collaboration (director, TD, camera) in shared scenes;Evaluation plans that combine presence/usability with scenario outcomes.


## 7. Future Perspectives and Conclusions

As immersive technologies and artificial intelligence continue to evolve [81], their convergence in the context of media production education presents both remarkable opportunities and pressing challenges. Future developments must move toward more transparent, explainable [82,83], and ethically grounded AI systems [41] that not only personalize instruction but also justify their pedagogical choices. Equally important is the integration of educators into the loop, enabling AI systems to coordinate with human judgment rather than replace it. Viewed through the lens of Learning with Human Feedback, teacher-in-the-loop orchestration offers a concrete and scalable pathway for aligning adaptive AI behavior with expert pedagogical judgment, enabling continuous improvement without displacing human authority. Such teacher-in-the-loop designs will ensure that adaptive learning remains pedagogically meaningful and contextually appropriate. Overall, most included studies were assessed as medium-quality, with a smaller proportion rated as high-quality and a limited number as low-quality, reflecting the exploratory and design-oriented nature of the field. To ensure consistency with the Abstract and the Results, we emphasize that our corpus-level conclusions are drawn from the synthesized evidence across the included studies (2013–2024) identified via the PRISMA 2020-aligned screening process. Given the heterogeneity of interventions and outcomes, we conducted a narrative synthesis complemented by descriptive statistics; therefore, no meta-analysis was performed. We report counts and proportions of AI components and interaction modalities, summarize commonly used evaluation instruments, and relate design choices to educational outcomes where comparable. No new human participant data were collected by this review.

Emotion-aware and neuroadaptive systems represent another promising frontier. By detecting learners’ affective and cognitive states, future platforms can adjust task difficulty, pace, and modality to sustain engagement and reduce frustration. The incorporation of generative AI [84,85,86], such as large language models [87,88,89,90] and diffusion-based tools, offers dynamic content generation capabilities—ranging from automated scene construction to interactive narrative scripting—thus expanding opportunities for creative exploration and feedback-rich practice [91].

However, realizing these possibilities demands robust evaluation frameworks that can assess not only task performance but also creative growth, emotional resilience, and collaborative skill development. Cross-cultural and distributed VR learning platforms may further enrich media education [92] by fostering global collaboration and diverse aesthetic expression.

Recent advances in generative AI and intelligent software engineering further strengthen the motivation for treating VR training content development as an integrated production workflow rather than a collection of isolated tools. In modern software systems, context-aware AI copilots combined with retrieval-augmented generation (RAG) have demonstrated how code generation, testing, and deployment can be unified within CI/CD-style pipelines, significantly reducing development overhead while improving robustness and scalability [93]. This paradigm is highly relevant to VR training and media production, where heterogeneous assets (e.g., 3D scenes, interaction logic, analytics, and assessment modules) must be created, managed, and iteratively refined across multidisciplinary teams. These insights reinforce the need for intelligent, workflow-oriented software toolchains to support the next generation of AI-enhanced VR education systems.

In conclusion, this review has outlined how the integration of AI into virtual reality systems can transform media production education into a more adaptive, engaging, and scalable experience [94]. By centering learners and enabling real-time interaction, smart VR systems support the development of both technical and creative competencies critical to the demands of the media industry [95]. The future of this field will rely on interdisciplinary collaboration among educators, technologists, and cognitive scientists to design intelligent learning systems that are not only innovative but also equitable, explainable, and human-centered.

## Figures and Tables

**Table 1:** Human-centered learning principles applied to VR media production education.

Principle	Theoretical Basis	Application in VR Design	Media-Production Example
Embodied Cognition	[40,41]	Motion-based interaction; spatial manipulation	Gesture-controlled camera blocking; light-stand positioning in 3D sets
Learner Agency	[42,43]	Free navigation; branching scenarios; sandbox tasks	Director chooses shot-list order and rehearses alternatives
Presence & Flow	[38,39]	High-fidelity scenes; minimal UI friction	Realistic control room with low-latency switching practice
Affective Feedback	[44,45]	Emotion-adaptive prompts and pacing	Stress-aware rehearsal timing during live-broadcast simulation
Social Constructivism	[28,46]	Multi-user co-presence; mentor avatars	Team roles (director, TD, camera) rehearsed in shared VR studio

**Table 2:** Key AI components in smart VR learning systems and typical uses in media training.

AI Technique	Primary Function	Typical Use in VR Media Training
Learner Modeling	Profile learner state	Adjusts task difficulty, tool availability, and feedback timing based on interaction logs and progression
Emotion Recognition	Detect affective state	Triggers offloading prompts or motivational nudges; escalates challenge under high engagement
Reinforcement Learning	Optimize learning path	Learns scenario sequencing and scaffolded exercises for editing/directing tasks
NLP Dialogue Agents	Conversational guidance	Answers “why/how” questions; context-aware critique of pacing or lighting
Generative AI	Scenario/content generation	Creates scripts, shot lists, or visual inserts aligned with learning objectives

**Table 3:** Evaluation dimensions and instruments commonly used in smart VR learning systems, with reporting tips.

Dimension	Metric/Indicator	Method/Tool	Reporting Tips
Engagement	Time-on-task; fixations; scene switches	Eye tracking (e.g., Tobii); interaction logs	Report sampling rate; AOI definition; smoothing/windowing rules.
Learning Effectiveness	Pre/post gains; task accuracy	Scenario-based tests; domain rubrics	Report effect direction with mean, SD, *n*; note task fidelity/rubric.
Affective Experience	HRV; frustration detection	Biofeedback; affect models	State sensor placement; window length; features (e.g., RMSSD); classifier type.
Usability	SUS [78]	Questionnaire; interviews	Report SUS mean and distribution; summarize major open-coded themes.
Collaboration	Turn-taking; coordination	Audio logs; NLP dialogue analysis	Define coding scheme; report inter-rater reliability for discourse labels.

## Data Availability

No new primary data were collected in this study. All analyses were conducted exclusively on previously published and publicly available literature.

## Supplementary Materials

The following supporting information can be downloaded at: https://www.mdpi.com/article/10.3390/biomimetics11010034/s1 (PRISMA 2020 checklist).

## Abbreviations

AI	Artificial Intelligence
VR	Virtual Reality
AR	Augmented Reality
HITL	Human-in-the-Loop
RL	Reinforcement Learning
NLP	Natural Language Processing
LLM	Large Language Model
EEG	Electroencephalography
PRISMA	Preferred Reporting Items for Systematic Reviews and Meta-Analyses
UI	User Interface
UX	User Experience
