# Supplementary material for: Toward Smart VR Education in Media Production: Integrating AI into Human-Centered and Interactive Learning Systems
Source: Biomimetics (Basel). 2026 Jan 4;11(1):34. doi: 10.3390/biomimetics11010034 (PMC12838545; doi:10.3390/biomimetics11010034)
Supplement: Supplementary file 1 [file biomimetics-11-00034-s001.zip › biomimetics-4035329-supplementary.pdf]

PRISMA 2020 Checklist

Section and Topic	Item #	Checklist item	Location where item is reported
**TITLE**			
Title	1	Identify the report as a systematic review.	Title explicitly identifies the study as a systematic review (“Toward Smart VR Education in Media Production: Integrating AI into Human-Centered and Interactive Learning Systems”).
**ABSTRACT**			
Abstract	2	See the PRISMA 2020 for Abstracts checklist.	Abstract reports objectives, information sources, study selection, synthesis approach, main findings, and implications, consistent with PRISMA 2020 for Abstracts.
**INTRODUCTION**			
Rationale	3	Describe the rationale for the review in the context of existing knowledge.	Introduction (Section 1) describes the rationale by identifying gaps in existing VR and AI education literature and the lack of domain-specific reviews for media production education.
Objectives	4	Provide an explicit statement of the objective(s) or question(s) the review addresses.	Objectives clearly stated in Introduction (Section 1) and reiterated at the end of Section 1, outlining the aim to systematically synthesize AI-integrated VR systems for media production education.
**METHODS**			
Eligibility criteria	5	Specify the inclusion and exclusion criteria for the review and how studies were grouped for the syntheses.	Eligibility criteria reported in Section 2.2 (Inclusion and Exclusion Criteria), specifying study type, domain relevance, design features, and language restrictions.
Information sources	6	Specify all databases, registers, websites, organisations, reference lists and other sources searched or consulted to identify studies. Specify the date when each source was last searched or consulted.	Information sources described in Section 2.1, including Scopus, Web of Science, IEEE Xplore, ACM Digital Library, and SpringerLink; search period 2013–2024. All databases were last searched in December 2024.
Search strategy	7	Present the full search strategies for all databases, registers and websites, including any filters and limits used.	Full search strategies, including Boolean operators, keyword clusters, and a representative canonical query, are reported in Section 2.1 (Search Strategy).
Selection process	8	Specify the methods used to decide whether a study met the inclusion criteria of the review, including how many reviewers screened each record and each report retrieved, whether they worked independently, and if applicable, details of automation tools used in the process.	Selection process detailed in Section 2.3. Title/abstract and full-text screening conducted independently by two reviewers, with disagreements resolved by discussion or a third reviewer.
Data collection process	9	Specify the methods used to collect data from reports, including how many reviewers collected data from each report, whether they worked independently, any processes for obtaining or confirming data from study investigators, and if applicable, details of automation tools used in the process.	Data extraction procedures described in Section 2.4, including use of a structured codebook and independent coding by two reviewers.
Data items	10a	List and define all outcomes for which data were sought. Specify whether all results that were compatible with each outcome domain in each study were sought (e.g. for all measures, time points, analyses), and if not, the methods used to decide which results to collect.	Outcome domains defined in Section 2.4, including usability, presence, workload, affect, collaboration, and learning performance.
	10b	List and define all other variables for which data were sought (e.g. participant and intervention characteristics, funding sources). Describe any assumptions made about any missing or unclear information.	Additional variables such as VR hardware, interaction modalities, AI components, instructional context, and data sources described in Section 2.4.
Study risk of bias	11	Specify the methods used to assess risk of bias in the included	Quality appraisal methods reported in Section 2.5, including criteria on study design

PRISMA

PRISMA 2020 Checklist

Section and Topic	Item #	Checklist item	Location where item is reported
assessment		studies, including details of the tool(s) used, how many reviewers assessed each study and whether they worked independently, and if applicable, details of automation tools used in the process.	clarity, measurement validity, and bias control; assessments conducted independently by two reviewers.
Effect measures	12	Specify for each outcome the effect measure(s) (e.g. risk ratio, mean difference) used in the synthesis or presentation of results.	Effect measures not quantitatively synthesized; narrative synthesis and descriptive statistics reported as stated in Section 2.6.
Synthesis methods	13a	Describe the processes used to decide which studies were eligible for each synthesis (e.g. tabulating the study intervention characteristics and comparing against the planned groups for each synthesis (item #5)).	Processes for grouping and synthesizing studies described in Sections 2.4 and 2.6.
	13b	Describe any methods required to prepare the data for presentation or synthesis, such as handling of missing summary statistics, or data conversions.	Data preparation and coding procedures reported in Section 2.4.
	13c	Describe any methods used to tabulate or visually display results of individual studies and syntheses.	Results presented using tables and figures (Figures 1–7; Tables 1–4) as described throughout Sections 3–5.
	13d	Describe any methods used to synthesize results and provide a rationale for the choice(s). If meta-analysis was performed, describe the model(s), method(s) to identify the presence and extent of statistical heterogeneity, and software package(s) used.	Narrative synthesis approach justified in Section 2.6 due to heterogeneity of interventions and outcomes; no meta-analysis conducted.
	13e	Describe any methods used to explore possible causes of heterogeneity among study results (e.g. subgroup analysis, meta-regression).	Conceptual exploration of heterogeneity discussed across Sections 4 and 6; no statistical heterogeneity analysis performed.
	13f	Describe any sensitivity analyses conducted to assess robustness of the synthesized results.	Not applicable; sensitivity analyses were not conducted due to narrative synthesis design.
Reporting bias assessment	14	Describe any methods used to assess risk of bias due to missing results in a synthesis (arising from reporting biases).	Reporting bias considerations discussed qualitatively in Section 6 (Challenges and Limitations).
Certainty assessment	15	Describe any methods used to assess certainty (or confidence) in the body of evidence for an outcome.	Formal certainty grading not conducted; overall confidence discussed qualitatively in Sections 6 and 7.
**RESULTS**			
Study selection	16a	Describe the results of the search and selection process, from the number of records identified in the search to the number of studies included in the review, ideally using a flow diagram.	Study selection results reported in Section 2.3 and illustrated in PRISMA flow diagram (Figure 2).
	16b	Cite studies that might appear to meet the inclusion criteria, but which were excluded, and explain why they were excluded.	Reasons for exclusion at the full-text eligibility stage are summarized by category in Section 2.3 and visualized in the PRISMA flow diagram (Figure 2), including irrelevance to the review scope, unavailability of full text, and failure to meet design criteria.
Study characteristics	17	Cite each included study and present its characteristics.	Characteristics of included studies summarized across Sections 3 and 4 and in Tables 2–4.
Risk of bias in studies	18	Present assessments of risk of bias for each included study.	Quality appraisal outcomes discussed in Section 2.5 and reflected in interpretive synthesis.
Results of individual studies	19	For all outcomes, present, for each study: (a) summary statistics for each group (where appropriate) and (b) an effect estimate and its precision (e.g. confidence/credible interval), ideally using structured	Individual study findings are summarized narratively in Sections 4 and 5, focusing on reported outcomes, system characteristics, and illustrative examples. Study-level effect estimates and precision metrics were not consistently reported across primary studies

PRISMA

PRISMA 2020 Checklist

Section and Topic	Item #	Checklist item	Location where item is reported
		tables or plots.	and were therefore not synthesized.
Results of syntheses	20a	For each synthesis, briefly summarise the characteristics and risk of bias among contributing studies.	Synthesis results summarized thematically in Sections 4 and 5.
	20b	Present results of all statistical syntheses conducted. If meta-analysis was done, present for each the summary estimate and its precision (e.g. confidence/credible interval) and measures of statistical heterogeneity. If comparing groups, describe the direction of the effect.	Not applicable; no meta-analysis performed (Section 2.6).
	20c	Present results of all investigations of possible causes of heterogeneity among study results.	Qualitative discussion of heterogeneity across systems and evaluations in Sections 4 and 6.
	20d	Present results of all sensitivity analyses conducted to assess the robustness of the synthesized results.	Not applicable.
Reporting biases	21	Present assessments of risk of bias due to missing results (arising from reporting biases) for each synthesis assessed.	Potential reporting biases discussed qualitatively in Section 6.
Certainty of evidence	22	Present assessments of certainty (or confidence) in the body of evidence for each outcome assessed.	Overall certainty and limitations of evidence discussed in Sections 6 and 7.
**DISCUSSION**			
Discussion	23a	Provide a general interpretation of the results in the context of other evidence.	General interpretation of results provided in Sections 5 and 7.
	23b	Discuss any limitations of the evidence included in the review.	Limitations of included evidence discussed in Section 6.
	23c	Discuss any limitations of the review processes used.	Review-level limitations discussed in Section 6.
	23d	Discuss implications of the results for practice, policy, and future research.	Implications for practice, policy, and future research discussed in Sections 6 and 7.
**OTHER INFORMATION**			
Registration and protocol	24a	Provide registration information for the review, including register name and registration number, or state that the review was not registered.	This systematic review was not registered.
	24b	Indicate where the review protocol can be accessed, or state that a protocol was not prepared.	A review protocol was not prepared.
	24c	Describe and explain any amendments to information provided at registration or in the protocol.	Not applicable.
Support	25	Describe sources of financial or non-financial support for the review, and the role of the funders or sponsors in the review.	Funding sources reported in the Funding section.
Competing interests	26	Declare any competing interests of review authors.	Competing interests declared in the Conflicts of Interest section.
Availability of data, code and other materials	27	Report which of the following are publicly available and where they can be found: template data collection forms; data extracted from included studies; data used for all analyses; analytic code; any other materials used in the review.	Data Availability Statement reports that no new data were created or analyzed.

PRISMA 2020 Checklist

From: Page MJ, McKenzie JE, Bossuyt PM, Boutron I, Hoffmann TC, Mulrow CD, et al. The PRISMA 2020 statement: an updated guideline for reporting systematic reviews. BMJ 2021;372:n71. doi: 10.1136/bmj.n71. This work is licensed under CC BY 4.0. To view a copy of this license, visit https://creativecommons.org/licenses/by/4.0/
